# Nicotinamide Riboside Attenuates Cisplatin-Induced Hepatorenal Toxicity Through Restoration of NAD^+^ Homeostasis and Nrf2/NQO1-Dependent Antioxidant Signaling

**DOI:** 10.3390/cells15161436

**Published:** 2026-08-10

**Authors:** Waleed Khaled Younis Albahadly, Mohammed Ibrahim Rasool, Haider Falih Shamikh Al-Saedi, Zahraa Abed Al-Kareem, Samer Ali Hasan, Mohammed Abdulaali Sahib, Meeqaat H. ALtrufi

**Affiliations:** 1 Department of Pharmacology and Toxicology, College of Pharmacy, University of Kerbala, Karbala 56001, Iraq; mohammed.i@uokerbala.edu.iq (M.I.R.); zahraa.alrikaby@uokerbala.edu.iq (Z.A.A.-K.); 2 Department of Pharmacology and Toxicology, College of Pharmacy, University of Al-Ameed, Karbala 56001, Iraq; hasaedi@alameed.edu.iq; 3 Department of Pharmaceutical Chemistry, Faculty of Pharmacy, University of Kufa, Najaf 54001, Iraq; samra.hasan@uokufa.edu.iq; 4 Department of Pharmaceutical Chemistry, College of Pharmacy, University of Kerbala, Karbala 56001, Iraq; mohammed.a.sahib@uokerbala.edu.iq; 5 Department of Pharmaceutical Chemistry, Faculty of Pharmacy, Ahl Al Bayt University Kerbala, Karbala 56001, Iraq; meeqaath.alturfi@student.uokufa.edu.iq

**Keywords:** nicotinamide riboside, cisplatin, hepatorenal toxicity, oxidative stress, antioxidant response, gene expression, Nrf2, NQO1, histopathology

## Abstract

Background: Cisplatin is a widely used chemotherapeutic agent whose clinical application is often limited by severe hepatorenal toxicity associated with oxidative stress and cellular injury. Nicotinamide riboside (NR), a natural precursor of nicotinamide adenine dinucleotide (NAD+), has emerged as a promising cytoprotective compound with antioxidant and metabolic regulatory properties. This study investigated the protective effects of NR against cisplatin-induced hepatorenal toxicity and explored its potential mechanisms of action. Methods: Thirty-six adult male Wistar rats were randomly assigned to four groups (*n* = 6): control, cisplatin (7 mg/kg, i.p.), nicotinamide riboside (50 mg/kg/day, orally), and cisplatin plus NR. Renal and hepatic function biomarkers, lipid profile parameters, oxidative stress markers, and antioxidant status were evaluated. Relative mRNA expression of Nrf2 and NQO1 was determined using RT-qPCR. Histopathological examinations of liver and kidney tissues were also performed. Results: Cisplatin administration induced marked hepatorenal injury, evidenced by significant elevations in serum KIM-1 (395.27 vs. 116.04 ng/mL), urea (71.16 vs. 21.33 mg/dL), creatinine (3.49 vs. 0.26 mg/dL), AST (325.83 vs. 95.16 U/L), and ALT (102.83 vs. 45.50 U/L), accompanied by dyslipidemia, oxidative stress, and severe histopathological alterations. NR treatment significantly attenuated these changes, reducing KIM-1, urea, creatinine, AST, and ALT by 55.5%, 47.8%, 48.1%, 55.4%, and 33.5%, respectively, compared with the cisplatin group. NR also improved antioxidant status by increasing GSH and SOD levels while reducing MDA and NO concentrations. Hepatic NAD^+^ levels and the NAD^+^/NADH ratio were significantly decreased by cisplatin and significantly restored by NR treatment. In addition, NR significantly upregulated the relative mRNA expression of Nrf2 and NQO1 and markedly preserved hepatic and renal histological architecture. Pharmacological inhibition of Nrf2 with ML385 significantly attenuated these protective effects of NR across biochemical, lipid, and oxidative stress parameters, confirming that they are, at least in part, Nrf2-dependent. Conclusions: Nicotinamide riboside exerts protective effects against cisplatin-induced hepatorenal toxicity that are mechanistically linked to activation of the Nrf2/NQO1 antioxidant pathway and restoration of hepatic NAD^+^ homeostasis. This finding supports the potential of NR as an adjunctive strategy for mitigating cisplatin-associated hepatorenal injury and warrants further preclinical and clinical investigation.

## 1. Introduction

Cisplatin (cis-diamminedichloroplatinum II) is one of the most effective platinum-based chemotherapeutic agents used for the treatment of various solid malignancies, including lung, ovarian, bladder, and testicular cancers. However, its clinical utility is frequently limited by severe dose-dependent toxicities, particularly nephrotoxicity and hepatotoxicity. Increasing evidence indicates that oxidative stress, mitochondrial dysfunction, inflammation, and dysregulated cellular responses are major contributors to cisplatin-induced tissue injury and organ dysfunction [1,2].

Excessive generation of reactive oxygen species (ROS) following cisplatin administration disrupts cellular redox homeostasis, leading to lipid peroxidation, DNA damage, mitochondrial dysfunction, and cellular injury [3,4]. Nuclear factor erythroid 2-related factor 2 (Nrf2) is a key regulator of cellular antioxidant defense. Upon activation, Nrf2 translocates to the nucleus and promotes the transcription of several cytoprotective genes, including NAD(P)H quinone oxidoreductase-1 (NQO1) and other antioxidant enzymes that contribute to the maintenance of redox balance. Impaired Nrf2 signaling has been strongly implicated in the pathogenesis of cisplatin-induced oxidative damage [5,6].

Nicotinamide riboside (NR), a naturally occurring precursor of nicotinamide adenine dinucleotide (NAD+), has attracted considerable attention because of its antioxidant, anti-inflammatory, and mitochondrial protective properties. By increasing intracellular NAD+ availability, NR enhances mitochondrial function, improves cellular bioenergetics, and promotes adaptive cellular stress responses. Previous studies have demonstrated that NR improves mitochondrial bioenergetics and modulates antioxidant pathways associated with Nrf2 signaling [7,8,9,10,11]. Moreover, toxicological and preclinical investigations have consistently demonstrated that NR is well tolerated and exhibits a favorable safety profile at pharmacologically relevant doses, supporting its potential for long-term therapeutic applications [12,13].

Although previous studies have reported the beneficial effects of NR in various models of oxidative stress and mitochondrial dysfunction, and a limited number of investigations have explored its protective effects against cisplatin-induced complications, the mechanisms underlying its ability to attenuate simultaneous hepatic and renal injury remain incompletely understood. In particular, further evidence is needed regarding its effects on oxidative stress modulation, antioxidant defense systems, and cytoprotective gene expression during cisplatin-induced hepatorenal toxicity.

We therefore hypothesized that nicotinamide riboside protects against cisplatin-induced hepatorenal injury by restoring redox homeostasis, enhancing endogenous antioxidant defenses, and modulating cytoprotective gene expression. Accordingly, the present study was designed to evaluate the protective effects of nicotinamide riboside against cisplatin-induced hepatorenal toxicity in rats through comprehensive biochemical, molecular, and histopathological analyses.

## 2. Materials and Methods

### 2.1. Chemicals and Reagents

Cisplatin (≥99% purity) and nicotinamide riboside (NR) were purchased from Sigma-Aldrich (St. Louis, MO, USA). TRIzol reagent was obtained from Invitrogen (Carlsbad, CA, USA). SYBR Green Master Mix and reverse transcription kits were purchased from Thermo Fisher Scientific (Waltham, MA, USA). ELISA kits were obtained from Elabscience Biotechnology Co., Ltd. (Wuhan, China). All chemicals used were of analytical grade.

### 2.2. Experimental Design

36 healthy adult male Wistar rats (11–12 weeks old, weighing 150–200 g) were housed under standard laboratory conditions (22 ± 2 °C, 50–60% humidity, 12 h light/dark cycle) with free access to food and water. Animals were acclimatized for one week before experimentation according to the Guide for the Care and Use of Laboratory Animals [12].

Rats were randomly assigned into six experimental groups (*n* = 6 per group):

Group I (Control): Animals received normal saline.

Group II (Cisplatin): Animals received a single intraperitoneal injection of cisplatin (7 mg/kg).

Group III (Cisplatin + NR): Animals received cisplatin (7 mg/kg, i.p.) and nicotinamide riboside (50 mg/kg/day, orally) for 15 consecutive days.

Group IV (NR): Animals received nicotinamide riboside (50 mg/kg/day, orally) for 15 consecutive days.

Group V (Cisplatin + ML385): Animals received cisplatin (7 mg/kg, i.p.) and ML385 (30 mg/kg/day, i.p.).

Group VI (Cisplatin + NR + ML385): Animals received cisplatin (7 mg/kg, i.p.), nicotinamide riboside (50 mg/kg/day, orally), and ML385 (30 mg/kg/day, i.p.). ML385 was administered 30 min before NR throughout the treatment period [13,14].

### 2.3. Biochemical Analysis

Serum kidney injury molecule-1 (KIM-1) levels were measured using a commercially available ELISA kit (SunLong Biotech Co., Ltd., Hangzhou, China; Cat. No. SL0433Ra).

Determination of Serum Urea: Serum urea levels were determined using a commercial diagnostic kit (Spectrum Diagnostics, Obour City, Egypt; Cat. No. 250001) according to the manufacturer’s instructions. The assay was based on the enzymatic urease–Berthelot colorimetric method, with absorbance measured at 578 nm.

Determination of Serum Creatinine: Serum creatinine levels were determined using a commercial diagnostic kit (Spectrum Diagnostics, Obour City, Egypt; Cat. No. 234001) according to the manufacturer’s instructions. The assay was based on the kinetic Jaffe method, with absorbance measured at 492 nm.

Serum alanine aminotransferase (ALT) and aspartate aminotransferase (AST) activities were determined using commercial diagnostic kits (Spectrum Diagnostics, Obour City, Egypt; Cat. No. 265001 for ALT and Cat. No. 261001 for AST) following the recommendations of the International Federation of Clinical Chemistry (IFCC).

Serum lipid profile parameters, including total cholesterol (TC), triglycerides (TGs), low-density lipoprotein cholesterol (LDL-C), and high-density lipoprotein cholesterol (HDL-C), were determined using enzymatic colorimetric kits (Pars Azmun Co., Tehran, Iran; Lot No. 97003 for TC, 97002 for TG, 97001 for LDL-C, and 97011 for HDL-C) according to the manufacturer’s instructions and standard laboratory procedures [15].

### 2.4. Assessment of Oxidative Stress Markers

Determination of Malondialdehyde (MDA): Hepatic and renal malondialdehyde (MDA) levels were determined using a commercially available ELISA kit (SunLong Biotech Co., Ltd., Hangzhou, China; Cat. No. SL0136Ra) according to the manufacturer’s instructions. The assay is based on a sandwich enzyme-linked immunosorbent assay (ELISA), with absorbance measured at 450 nm using a microplate reader.

Determination of Reduced Glutathione (GSH): Hepatic and renal reduced glutathione (GSH) levels were determined using a commercially available ELISA kit (SunLong Biotech Co., Ltd., Hangzhou, China; Cat. No. SL1935Ra) according to the manufacturer’s instructions. The assay is based on a sandwich ELISA, with absorbance measured at 450 nm using a microplate reader.

Determination of Nitric Oxide (NO): Nitric oxide (NO) concentrations were determined using a commercially available ELISA kit (SunLong Biotech Co., Ltd., Hangzhou, China; Cat. No. SL2137Ra) according to the manufacturer’s instructions. The assay is based on a sandwich ELISA, with absorbance measured at 450 nm using a microplate reader.

Determination of Superoxide Dismutase (SOD) Activity: Superoxide dismutase (SOD) activity was determined using a commercially available ELISA kit (SunLong Biotech Co., Ltd., Hangzhou, China; Cat. No. SL2245Ra) according to the manufacturer’s instructions.

Determination of Hepatic NAD^+^, NADH, and NAD^+^/NADH Ratio: Hepatic NAD^+^ and NADH levels were determined using a commercially available NAD/NADH Assay Kit (Sigma-Aldrich, St. Louis, MO, USA; Catalog No. MAK468) according to the manufacturer’s instructions. Briefly, immediately after euthanasia, liver tissues were rapidly excised, rinsed with ice-cold phosphate-buffered saline (PBS) to remove residual blood, and maintained on ice. Approximately 20–50 mg of liver tissue was homogenized in the NAD/NADH extraction buffer supplied with the kit. The homogenates were centrifuged at 12,000× *g* for 5 min at 4 °C, and the resulting supernatants were collected for subsequent analysis. NAD^+^ and NADH concentrations were determined using the enzymatic cycling colorimetric assay provided in the kit. Absorbance was measured at 565 nm using a microplate reader. The concentrations of NAD^+^ and NADH were calculated from a standard curve generated with the supplied NAD standard, and the NAD^+^/NADH ratio was subsequently calculated according to the manufacturer’s protocol.

### 2.5. RNA Extraction and Quantitative RT-PCR

Total RNA was extracted from liver and kidney tissues using TRIzol reagent (Invitrogen, USA) according to the manufacturer’s instructions and the method described by Chomczynski and Sacchi [16,17,18,19]. RNA concentration and purity were evaluated using a NanoDrop spectrophotometer (Thermo Scientific, USA), and only samples with A260/A280 ratios between 1.8 and 2.0 were used for subsequent analysis. Complementary DNA (cDNA) was synthesized using a reverse transcription kit (Thermo Fisher Scientific, USA). Quantitative real-time PCR (RT-qPCR) was performed using SYBR Green Master Mix on a Bio-Rad CFX96 Real-Time PCR Detection System (Bio-Rad, Hercules, CA, USA). The thermal cycling conditions consisted of an initial denaturation at 95 °C for 5 min, followed by 40 cycles of denaturation at 95 °C for 15 s, annealing at 60 °C for 30 s, and extension at 72 °C for 30 s. Relative mRNA expression levels of nuclear factor erythroid 2-related factor 2 (Nfe2l2) and NAD(P)H quinone oxidoreductase 1 (Nqo1) were quantified in both liver and kidney tissues using the comparative 2^−ΔΔCt method and normalized against the housekeeping genes GAPDH and HPRT1 [20]. Gene-specific primers were designed using the Primer-BLAST tool (version 2.5.0) provided by the National Center for Biotechnology Information (NCBI) [21].

### 2.6. Histopathological Examination

Kidney and liver tissues were fixed in 10% neutral buffered formalin, embedded in paraffin, sectioned at 5 μm, and stained with hematoxylin and eosin (H&E). Histological evaluation was performed according to Bancroft and Gamble [22].

### 2.7. Statistical Analysis

Statistical analyses were performed using SPSS version 20.0 (IBM Corp., Armonk, NY, USA). Differences among groups were analyzed using one-way ANOVA followed by Tukey’s post hoc multiple comparison test. A value of *p* < 0.05 was considered statistically significant [23].

## 3. Results

### 3.1. Effect of Nicotinamide Riboside on Renal and Hepatic Function Biomarkers

Cisplatin administration caused marked renal dysfunction compared with the control group. Serum KIM-1 levels were significantly increased (395.27 ± 18.78 vs. 116.04 ± 1.99 ng/mL, *p* < 0.05), accompanied by marked elevations in serum urea and creatinine levels (71.16 ± 4.26 vs. 21.33 ± 0.88 mg/dL and 3.49 ± 0.20 vs. 0.26 ± 0.04 mg/dL, respectively), indicating severe impairment of renal function (Table 1). Likewise, hepatic injury was evidenced by significant increases in serum AST and ALT activities in the cisplatin-treated rats compared with controls (325.83 ± 40.21 vs. 95.16 ± 33.41 U/L and 102.83 ± 13.96 vs. 45.50 ± 2.77 U/L, respectively).

Treatment with nicotinamide riboside significantly ameliorated these alterations. KIM-1, urea, creatinine, AST, and ALT levels were significantly reduced compared with the cisplatin group (*p* < 0.05). No significant differences were observed between the NR-alone and control groups (Table 2).

Cisplatin administration significantly increased serum KIM-1, urea, creatinine, AST, and ALT levels compared with the control group (*p* < 0.05), indicating severe renal and hepatic injury. Treatment with nicotinamide riboside (NR) significantly attenuated these alterations. Administration of the Nrf2 inhibitor ML385 markedly diminished the protective effects of NR, suggesting that the hepatorenal protective activity of NR is mediated, at least in part, through activation of the Nrf2 signaling pathway. 

### 3.2. Effect of Nicotinamide Riboside on Serum Lipid Profile

Cisplatin treatment induced significant dyslipidemia characterized by elevated serum total cholesterol, triglycerides, and LDL-C levels, together with a reduction in HDL-C compared with the control group (*p* < 0.05). Administration of nicotinamide riboside significantly improved lipid metabolism by decreasing total cholesterol, triglycerides, and LDL-C levels while partially restoring HDL-C levels compared with the cisplatin-treated animals. The NR-alone group exhibited lipid parameters comparable to those of the control group (Table 3).

As shown in Table 3, cisplatin administration significantly increased serum total cholesterol, triglycerides, and LDL-C levels while significantly decreasing HDL-C compared with the control group (*p* < 0.05), indicating marked dyslipidemia. Treatment with nicotinamide riboside (NR) significantly ameliorated these alterations by reducing total cholesterol, triglycerides, and LDL-C levels and increasing HDL-C compared with the cisplatin group (*p* < 0.05). In contrast, co-administration of the Nrf2 inhibitor ML385 markedly attenuated the beneficial effects of NR, resulting in a less pronounced improvement in the lipid profile. These findings suggest that the lipid-lowering effect of NR is mediated, at least in part, through activation of the Nrf2 signaling pathway. Data are presented as mean ± SD (*n* = 6). Means with different superscript letters (a–c) within the same column are significantly different (*p* < 0.05), whereas means sharing the same superscript letter are not significantly different. Bold values indicate statistically significant differences (*p* < 0.05).

### 3.3. Effect of Nicotinamide Riboside on Oxidative Stress and Antioxidant Status

Exposure to cisplatin resulted in pronounced oxidative stress, as demonstrated by significant reductions in hepatic GSH and SOD levels and concomitant elevations in MDA and NO concentrations compared with controls (*p* < 0.05). Treatment with nicotinamide riboside significantly restored antioxidant defenses by increasing GSH and SOD levels and reducing MDA and NO concentrations relative to the cisplatin group. The NR-alone group showed antioxidant parameters similar to those observed in the control group (Table 4).

As shown in Table 4, cisplatin administration significantly reduced hepatic GSH and SOD levels while significantly increasing MDA and NO levels compared with the control group (*p* < 0.05), indicating severe oxidative stress. Treatment with nicotinamide riboside (NR) significantly restored GSH and SOD levels and reduced MDA and NO levels compared with the cisplatin group (*p* < 0.05). In contrast, co-administration of the Nrf2 inhibitor ML385 markedly attenuated the antioxidant effects of NR, resulting in lower GSH and SOD levels and higher MDA and NO levels than those observed in the cisplatin + NR group. These findings indicate that the antioxidant effects of NR are mediated, at least in part, through activation of the Nrf2 signaling pathway. Data are presented as mean ± SD (*n* = 6). Means with different superscript letters (a–c) within the same column are significantly different (*p* < 0.05), whereas means sharing the same superscript letter are not significantly different. Bold values indicate statistically significant differences (*p* < 0.05).

### 3.4. Effect of Nicotinamide Riboside on Nrf2 and NQO1 mRNA Expression

**Figure 1:**
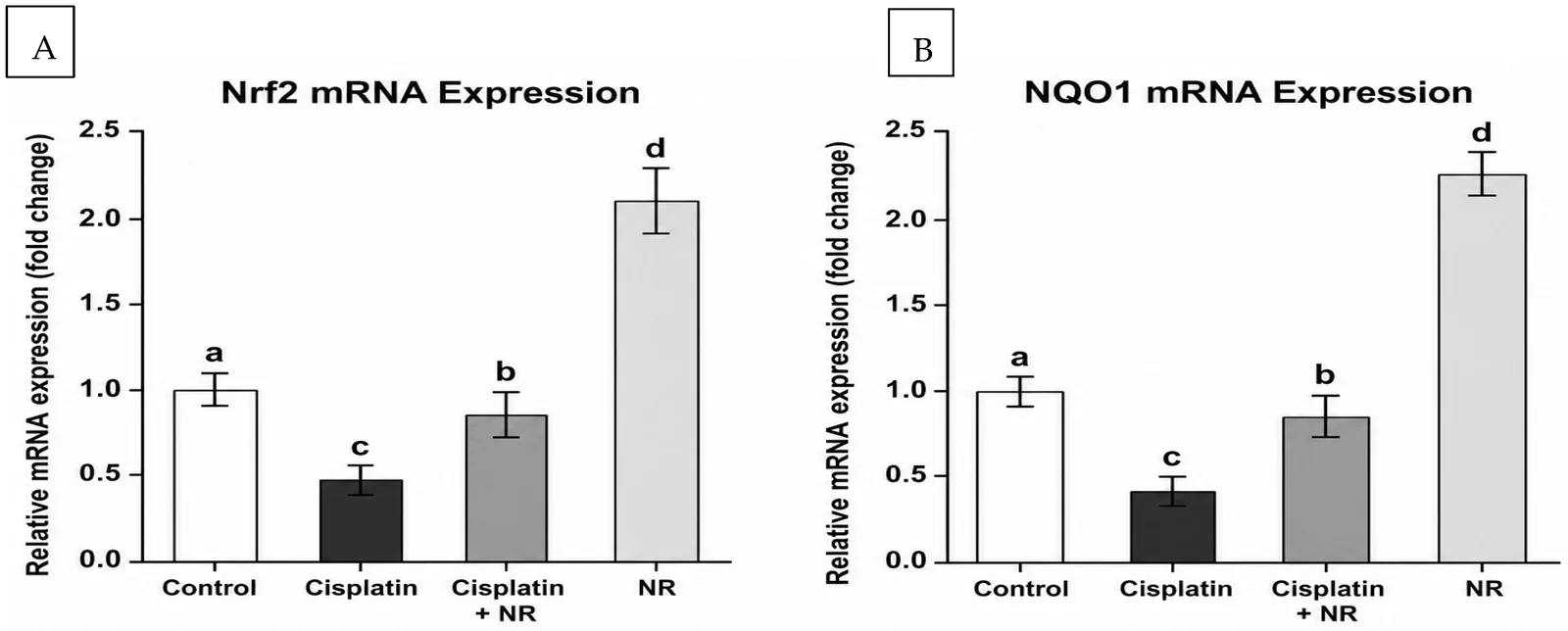
Relative mRNA expression levels of *Nrf2* (**A**) and *NQO1* (**B**) in liver tissues from different experimental groups. Total RNA was extracted from liver tissues, and gene expression was quantified by RT-qPCR using the 2^−ΔΔCt method. Expression levels were normalized to *GAPDH* and *HPRT1* and are presented as fold changes relative to the control group. Data are expressed as mean ± SD (*n* = 6). Statistical analysis was performed using one-way ANOVA followed by Tukey’s post hoc test. Bars with different letters indicate significant differences among groups (*p* < 0.05).

RT-qPCR analysis revealed a significant reduction in Nrf2 and NQO1 mRNA expression in the cisplatin-treated group compared with the control group (*p* < 0.05). Co-treatment with nicotinamide riboside significantly restored the expression of both genes. Moreover, the NR-alone group exhibited the highest expression levels of Nrf2 and NQO1, indicating activation of antioxidant signaling pathways and enhancement of cellular defense mechanisms against oxidative stress (Figure 1).

### 3.5. Effect of Nicotinamide Riboside on Hepatic NAD^+^, NADH, and NAD^+^/NADH Ratio

As shown in Table 5, cisplatin administration significantly decreased hepatic NAD^+^ levels and the NAD^+^/NADH ratio while increasing NADH levels compared with the control group (*p* < 0.05). Co-treatment with nicotinamide riboside (Cisplatin + NR) partially restored these parameters relative to the cisplatin group, although they did not completely return to control values. In contrast, the NR-alone group maintained hepatic NAD^+^ homeostasis, exhibiting higher NAD^+^ levels and a higher NAD^+^/NADH ratio than the cisplatin-treated groups. These findings provide direct biochemical evidence supporting enhanced NAD^+^ availability as a contributing mechanism underlying the protective effects of nicotinamide riboside.

As shown in Table 6, Data are presented as mean ± SD (*n* = 6 per group). Means with different superscript letters (a–c) within the same column are significantly different (*p* < 0.05), whereas means sharing the same superscript letter are not significantly different, as determined by one-way ANOVA followed by Tukey’s post hoc multiple comparison test. NAD^+^: oxidized nicotinamide adenine dinucleotide; NADH: reduced nicotinamide adenine dinucleotide.

### 3.6. Histopathological Findings

**Figure 2:**
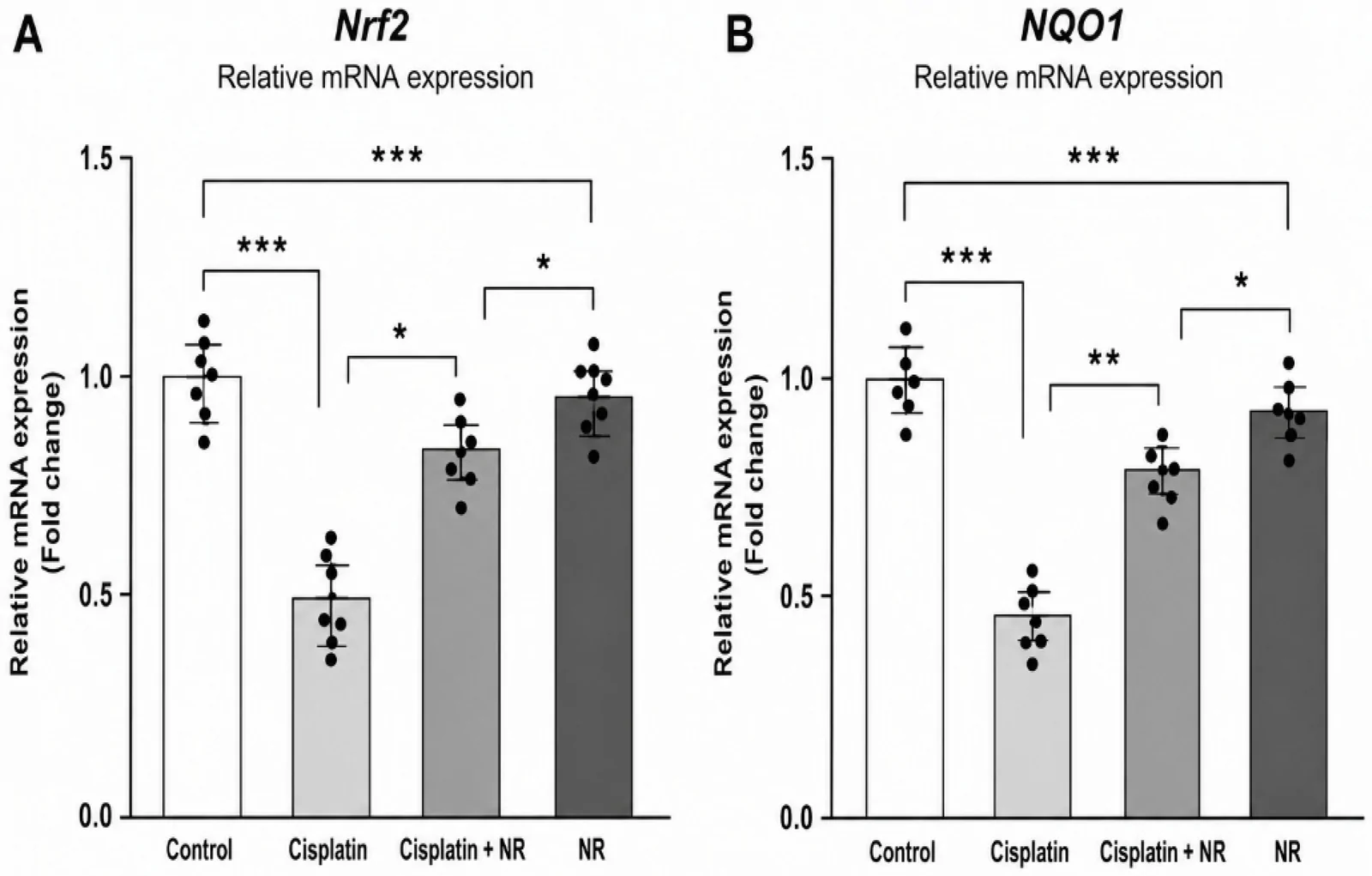
Relative mRNA expression of Nrf2 (**A**) and NQO1 (**B**) in kidney tissues from the different experimental groups. Total RNA was extracted from kidney tissues, and gene expression was quantified by quantitative real-time PCR (RT-qPCR) using the 2^−ΔΔCt method. Expression levels were normalized to the housekeeping genes GAPDH and HPRT1 and are presented as fold changes relative to the control group. Data are expressed as mean ± SD (*n* = 6). Statistical analysis was performed using one-way ANOVA followed by Tukey’s post hoc multiple comparison test. Asterisks indicate statistically significant differences (* *p* < 0.05, ** *p* < 0.01, *** *p* < 0.001).

Histopathological examination of liver sections from the control group revealed normal hepatic architecture with well-preserved hepatocytes and normal central veins. In contrast, liver sections from the cisplatin-treated group showed severe pathological alterations characterized by hepatocellular degeneration and necrosis, sinusoidal and vascular congestion, inflammatory cell infiltration, and disruption of the normal hepatic architecture. Treatment with nicotinamide riboside (NR) markedly ameliorated these lesions, as evidenced by reduced cellular degeneration and restoration of hepatic architecture. Liver tissues from the NR-alone group exhibited nearly normal histological features comparable to those observed in the control group. Similarly, kidney sections from the control group demonstrated normal glomeruli and intact renal tubules. However, the cisplatin-treated group exhibited marked tubular degeneration, epithelial vacuolation, vascular congestion, and glomerular injury. Co-administration of NR substantially attenuated these histopathological abnormalities and preserved renal architecture. Kidney sections from the NR-alone group showed normal glomerular and tubular structures similar to those of the control group (Figure 2).

## 4. Discussion

The present study provides comprehensive biochemical, molecular, and histopathological evidence demonstrating the protective effects of nicotinamide riboside (NR) against cisplatin-induced hepatorenal injury. Cisplatin administration caused significant renal and hepatic dysfunction, dyslipidemia, oxidative stress, and impairment of endogenous antioxidant defense mechanisms, accompanied by downregulation of Nrf2 and NQO1 mRNA expression and severe histopathological damage in hepatic and renal tissues. In contrast, NR treatment significantly improved biochemical parameters, restored antioxidant defenses, enhanced antioxidant gene expression, and preserved tissue architecture. Collectively, these findings indicate that NR exerts multi-target cytoprotective effects rather than acting solely as a conventional antioxidant [24,25,26,27]. While the antioxidant properties of NR and the involvement of Nrf2/NQO1 signaling in cisplatin toxicity have each been reported previously in isolation, the present work adds to this literature by directly linking restoration of hepatic NAD+/NADH status with concurrent recovery of Nrf2/NQO1 expression and preservation of hepatic and renal histoarchitecture within a single hepatorenal toxicity model, providing a more integrated biochemical-molecular-histological picture than studies addressing these endpoints separately.

**Figure 3:**
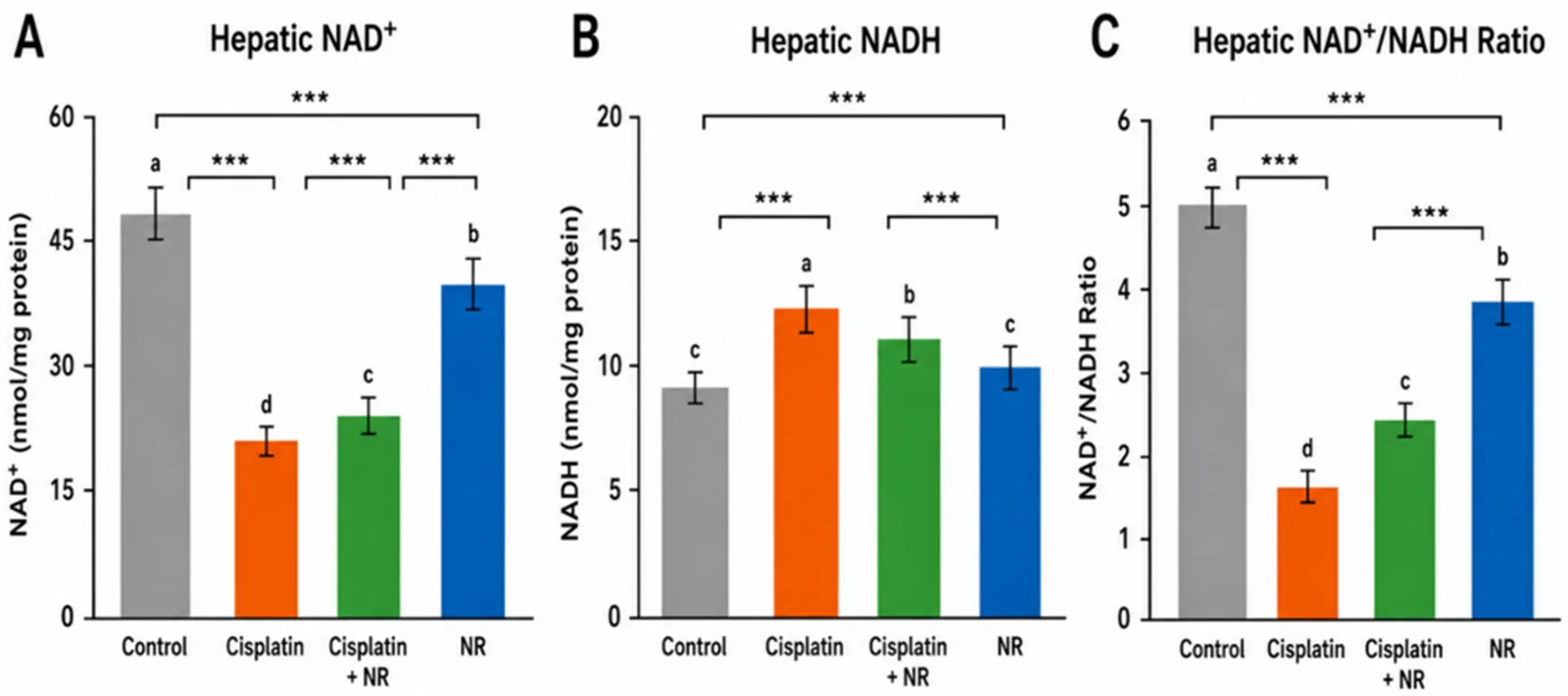
Effect of nicotinamide riboside (NR) on hepatic (**A**) NAD^+^ levels, (**B**) NADH levels, and (**C**) the NAD^+^/NADH ratio in rats. Cisplatin administration significantly decreased hepatic NAD^+^ levels and the NAD^+^/NADH ratio while increasing NADH levels compared with the control group (*p* < 0.05). Co-administration of nicotinamide riboside with cisplatin partially restored hepatic NAD^+^ levels and the NAD^+^/NADH ratio and reduced NADH levels compared with the cisplatin-treated group (*p* < 0.05). Administration of nicotinamide riboside alone maintained NAD^+^ homeostasis, showing higher NAD^+^ levels and NAD^+^/NADH ratio and lower NADH levels than the cisplatin-treated groups. Data are presented as mean ± SD (*n* = 6). Bars with different lowercase letters (a–d) indicate significant differences among groups (*p* < 0.05), whereas bars sharing the same letter are not significantly different. Statistical analysis was performed using one-way ANOVA followed by Tukey’s multiple comparison test. Asterisks indicate statistically significant differences (*** *p* < 0.001).

**Figure 4:**
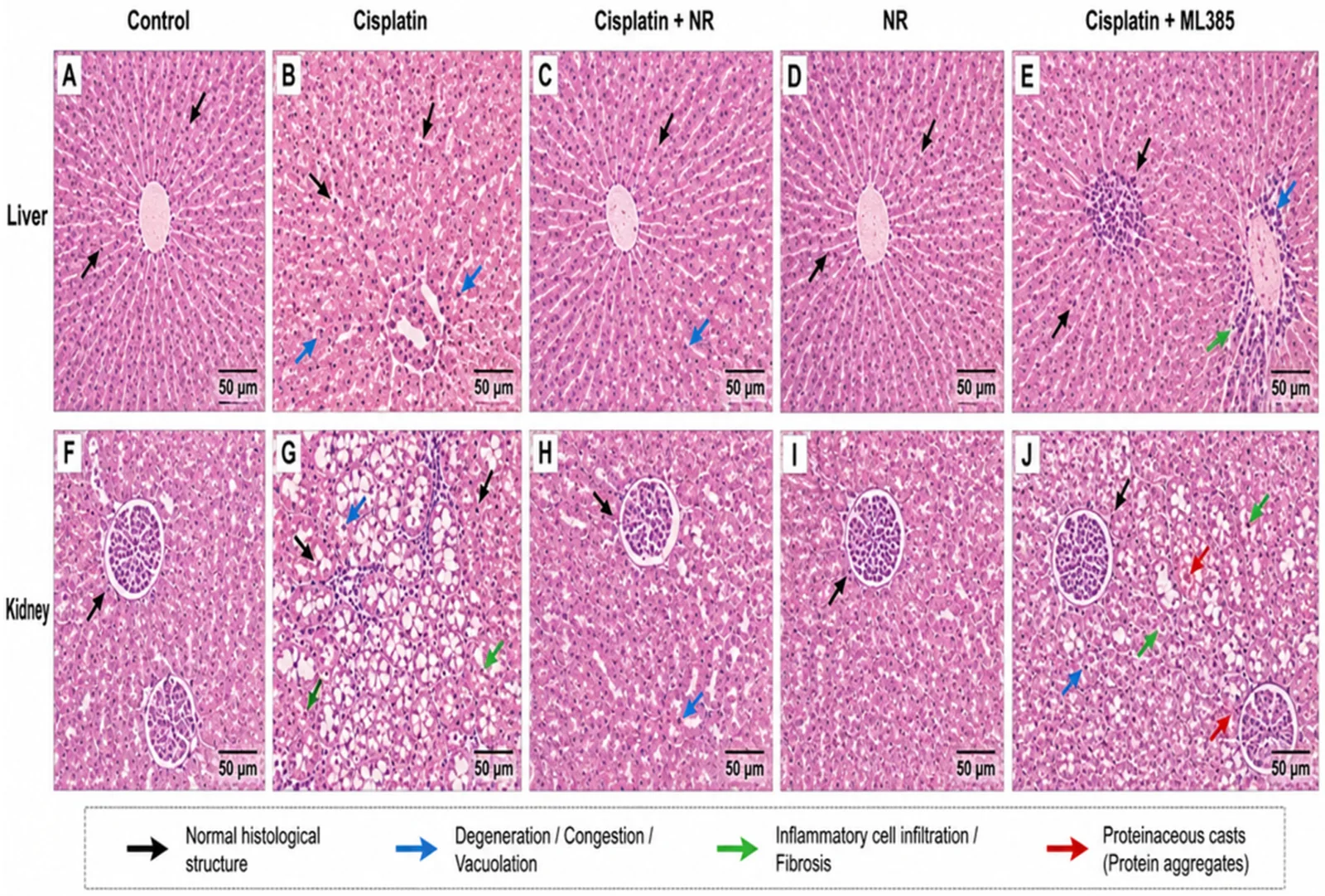
Histopathological examination of liver and kidney tissues stained with hematoxylin and eosin (H&E). (**A**) Liver section from the control group showing normal hepatic architecture with intact hepatocytes and central vein. (**B**) Liver section from the cisplatin-treated group showing hepatocellular degeneration and vascular congestion. (**C**) Liver section from the cisplatin + nicotinamide riboside (NR)-treated group showing marked improvement of hepatic architecture with only mild residual alterations. (**D**) Liver section from the NR-treated group showing normal hepatic histological architecture. (**E**) Kidney section from the control group showing normal glomeruli and renal tubules. (**F**) Kidney section from the cisplatin-treated group showing tubular degeneration, epithelial vacuolation, inflammatory cell infiltration, and vascular congestion. (**G**) Kidney section from the cisplatin + NR-treated group showing preservation of renal architecture with only mild pathological alterations. (**H**) Kidney section from the NR-treated group showing normal glomerular and tubular structures. (**I**) Liver section from the cisplatin + ML385-treated group showing centrilobular inflammation (black arrow), periportal inflammatory cell infiltration (blue arrow), and periportal fibrosis (green arrow). (**J**) Kidney section from the cisplatin + NR + ML385-treated group showing normal glomeruli (black arrow), vacuolar degeneration of proximal renal tubular epithelium (blue arrow), vacuolation of distal renal tubules (green arrow), and mild intratubular proteinaceous casts (protein aggregates) (red arrow). H&E stain; original magnification ×10 for panels (**I**,**J**) and ×40 for panels (**A**–**H**) (scale bar = 50 μm). Black arrows indicate normal histological structures; blue arrows indicate degeneration and/or vascular congestion; green arrows indicate inflammatory cell infiltration or fibrosis, as specified; red arrows indicate intratubular proteinaceous casts structures; blue arrows indicate degeneration/congestion; green arrows indicate inflammatory cell infiltration. Scale bars = 50 µm.

Oxidative stress is widely recognized as a central mechanism underlying cisplatin-induced organ toxicity. Excessive generation of reactive oxygen species (ROS) promotes lipid peroxidation, mitochondrial dysfunction, DNA damage, and activation of downstream inflammatory pathways [28,29]. Consistent with this mechanism, cisplatin-treated rats in the present study exhibited elevated MDA and nitric oxide levels alongside depletion of GSH and SOD, findings that align with previous reports linking impaired endogenous antioxidant defenses to cisplatin-induced renal and hepatic injury [28,30,31]. NR supplementation significantly restored antioxidant status, an effect consistent with improved mitochondrial redox homeostasis; since NR is an efficient NAD+ precursor, replenishment of intracellular NAD+ pools likely enhances mitochondrial function and increases resistance to oxidative insult. This was directly confirmed in the present study: hepatic NAD^+^ levels and the NAD^+^/NADH ratio were significantly reduced by cisplatin and significantly restored by NR treatment (Table 5; Figure 3 and Figure 4).

Mechanistically, NR is a naturally occurring NAD+ precursor with favorable oral bioavailability and tissue distribution. Following oral administration, NR is rapidly converted into nicotinamide mononucleotide (NMN) and subsequently NAD+, supporting multiple metabolic and cytoprotective processes. NAD+ depletion is increasingly recognized as a pivotal contributor to cisplatin-induced organ toxicity: oxidative stress and DNA damage activate NAD+-consuming enzymes, particularly poly(ADP-ribose) polymerases (PARPs), depleting intracellular NAD+ stores and impairing cellular bioenergetics, ATP production, and repair mechanisms, thereby exacerbating tissue injury. Restoration of NAD+ homeostasis through exogenous NAD+ precursors has therefore emerged as a promising therapeutic strategy across multiple experimental disease models [32]. As an essential cofactor for redox reactions in glycolysis, the tricarboxylic acid cycle, and oxidative phosphorylation, adequate NAD+ availability supports mitochondrial respiration and limits excessive ROS generation; NAD+-boosting interventions have been shown to improve mitochondrial quality control, enhance cellular stress resistance, and attenuate tissue damage under pathological conditions [13,33,34,35,36,37].

At the molecular level, activation of the Nrf2/NQO1 antioxidant axis represents one of the most important endogenous defense mechanisms against oxidative injury. Nrf2 governs the transcription of a broad array of cytoprotective genes involved in antioxidant defense and detoxification, while NQO1, a major downstream Nrf2 target, protects against oxidative damage by facilitating quinone detoxification and limiting ROS formation. The restoration of Nrf2 and NQO1 expression observed in the present study may therefore represent a key molecular mechanism underlying the protective effects of NR against cisplatin-induced hepatorenal injury [38], consistent with the broader pharmacokinetic and metabolic properties of NR described above [29,33,34,39,40]. A principal finding of this study was precisely this modulation of antioxidant-related gene expression: cisplatin markedly downregulated Nrf2 and NQO1 mRNA expression, consistent with impaired cellular antioxidant defense, and prior work has similarly linked suppressed Nrf2 signaling to oxidative stress and tissue injury during cisplatin exposure [41,42]. NR treatment significantly restored expression of both genes, indicating enhancement of endogenous antioxidant responses; together with the observed improvements in oxidative stress biomarkers and histopathological features, these molecular findings support a protective role for NR in cisplatin-induced hepatorenal injury [43,44]. Beyond oxidative stress, cisplatin-induced hepatorenal injury involves additional disturbances—including inflammation, metabolic dysfunction, and impaired cellular homeostasis—and the substantial improvements observed with NR across biochemical, oxidative stress, and histopathological parameters suggest broad cytoprotective effects attributable to enhanced NAD+ availability, supported energy metabolism, and strengthened endogenous antioxidant defenses [35,38].

Beyond this correlative molecular evidence, the present study provides direct mechanistic confirmation of Nrf2 involvement through pharmacological inhibition. Co-administration of the selective Nrf2 inhibitor ML385 markedly abolished the beneficial effects of NR on renal and hepatic function biomarkers, lipid profile, and oxidative stress parameters (Table 2, Table 3 and Table 4), despite the continued presence of NR. This loss of protection under Nrf2 blockade indicates that intact Nrf2 signaling is required for NR-mediated cytoprotection, rather than Nrf2 activation being merely a downstream consequence of reduced cellular injury. These findings establish a causal, rather than purely associative, link between Nrf2/NQO1 activation and the hepatorenal protective effects of NR, and are consistent with previous reports demonstrating that Nrf2 inhibition abrogates the cytoprotective actions of other antioxidant and NAD+-boosting compounds [42,43].

Histopathological findings provided strong morphological corroboration of the biochemical and molecular data. Liver sections from cisplatin-treated animals showed severe hepatocellular degeneration, necrosis, and vascular congestion, while kidney sections exhibited tubular degeneration, epithelial vacuolation, and glomerular congestion—classical features of cisplatin-induced hepatorenal toxicity described extensively in prior work. Co-administration of NR markedly attenuated these pathological changes and preserved near-normal hepatic and renal architecture [1,45,46,47], and the close concordance between histopathological findings and changes in oxidative stress markers and gene expression reinforces the protective role of NR. Collectively, these results indicate that modulation of oxidative stress and enhancement of antioxidant gene expression contribute substantially to the preservation of tissue integrity, positioning nicotinamide riboside as a promising adjunctive therapeutic strategy for minimizing cisplatin-induced hepatorenal toxicity [32,48]. Its capacity to restore redox homeostasis, enhance endogenous antioxidant defenses, improve biochemical and molecular parameters, and preserve histological architecture underscores its potential clinical relevance and warrants further investigation in translational and clinical studies [36,37,49].

### Study Limitations

Despite the promising findings of the present study, several limitations should be acknowledged. Pharmacological inhibition of Nrf2 with ML385 provided evidence that the protective effects of NR are, at least in part, Nrf2-dependent; however, ML385 is not perfectly Nrf2-selective, and off-target or Nrf2-independent contributions cannot be entirely excluded. Complementary approaches, such as Nrf2 gene-silencing or genetically modified (Nrf2-knockout) models, would further strengthen and refine this causal relationship. In addition, mitochondrial function was not directly evaluated in the present study. Although the observed improvements in oxidative stress markers and SOD levels may indirectly suggest enhanced mitochondrial homeostasis, parameters such as ATP production, mitochondrial membrane potential, oxygen consumption rate, and respiratory chain activity were not assessed. Furthermore, hepatic NAD+ and NADH concentrations and the NAD+/NADH ratio were directly measured across all four experimental groups and confirmed the restorative effect of NR on NAD+ status; however, broader NAD+-related metabolic pathways (e.g., PARP and sirtuin signaling) were not evaluated. Finally, molecular analyses were performed exclusively in liver tissue, whereas the mechanistic conclusions regarding renal protection were primarily supported by biochemical and histopathological findings. Therefore, future studies incorporating functional validation of Nrf2 signaling, direct assessment of mitochondrial bioenergetics, broader NAD+-related metabolic pathways, and mechanistic analyses in kidney tissue are warranted to further clarify the protective mechanisms of nicotinamide riboside against cisplatin-induced hepatorenal injury.

## 5. Conclusions

The present study provides comprehensive biochemical, molecular, and histopathological evidence demonstrating that nicotinamide riboside effectively attenuates cisplatin-induced hepatorenal injury. NR significantly improved renal and hepatic function, reducing serum KIM-1, urea, creatinine, AST, and ALT by 33–55% relative to the cisplatin group, while also restoring lipid homeostasis, reducing oxidative stress, and enhancing endogenous antioxidant defenses. These protective effects were associated with upregulation of Nrf2 and NQO1 gene expression, restoration of hepatic NAD^+^ homeostasis, and preservation of hepatic and renal histological architecture, suggesting that NR exerts multiple complementary cytoprotective effects rather than functioning solely as a conventional antioxidant. Importantly, pharmacological inhibition of Nrf2 with ML385 markedly abrogated these protective effects, providing direct mechanistic evidence that the hepatorenal protection conferred by NR is, at least in part, causally dependent on Nrf2/NQO1 pathway activation rather than a mere correlate of reduced injury. Therefore, NR may represent a promising adjunctive candidate for mitigating cisplatin-associated hepatorenal toxicity and improving the tolerability of platinum-based chemotherapy regimens. Further studies incorporating genetic validation of Nrf2 dependence, direct assessment of mitochondrial bioenergetics, broader NAD+-related metabolic pathways, and clinical investigations are warranted to confirm the underlying mechanisms and establish the translational potential of NR.

## Figures and Tables

**Table 1:** Primer sequences used for RT-qPCR analysis of liver and kidney tissues.

Gene Symbol	Gene Name	Forward Primer (5′–3′)	Reverse Primer (5′–3′)	Amplicon Size (bp)	Biological Function
Nfe2l2	Nuclear factor erythroid 2-related factor 2 (Nrf2)	GACCATGAGTCGCTTGC	AGAGCTATCGAGTGACTGAG	90	Master regulator of antioxidant defense
Nqo1	NAD(P)H quinone oxidoreductase 1	AGGATGGGAGGTACTCGAAT	TGCTAGAGATGACTCGGAAG	110	Phase II antioxidant enzyme
Gapdh	Glyceraldehyde-3-phosphate dehydrogenase	TGCACCACCAACTGCTTAGC	GGCATGGACTGTGGTCATGAG	120	Housekeeping gene
Hprt1	Hypoxanthine phosphoribosyltransferase 1	TCCTCCTCAGACCGCTTTT	CCTGGTTCATCATCGCTAATC	95	Housekeeping gene

**Table 2:** Effect of nicotinamide riboside (NR) and ML385 on renal and hepatic function biomarkers in cisplatin-treated rats.

Group	KIM-1 (ng/mL)	Urea (mg/dL)	Creatinine (mg/dL)	AST (U/L)	ALT (U/L)
Control	116.04 ± 1.99 ^c^	21.33 ± 0.88 ^c^	0.26 ± 0.04 ^c^	95.16 ± 33.41 ^c^	45.50 ± 2.77 ^c^
Cisplatin	395.27 ± 18.78 ^a^	71.16 ± 4.26 ^a^	3.49 ± 0.20 ^a^	325.83 ± 40.21 ^a^	102.83 ± 13.96 ^a^
Cisplatin + NR	175.86 ± 8.96 ^b^	37.16 ± 4.57 ^b^	1.81 ± 0.20 ^b^	145.16 ± 51.08 ^b^	68.33 ± 7.99 ^b^
NR	106.10 ± 1.99 ^c^	26.50 ± 4.20 ^c^	0.27 ± 0.05 ^c^	94.16 ± 46.70 ^c^	54.16 ± 5.47 ^c^
Cisplatin + ML385	360.50 ± 18.20 ^a^	65.20 ± 4.00 ^a^	3.10 ± 0.22 ^a^	300.40 ± 39.10 ^a^	96.50 ± 11.90 ^a^
Cisplatin + NR + ML385	255.30 ± 15.80 ^b^	49.80 ± 3.90 ^b^	2.35 ± 0.18 ^b^	210.60 ± 37.20 ^b^	80.40 ± 8.50 ^b^

Data are presented as mean ± SD (*n* = 6). Means with different superscript letters (a–c) within the same column are significantly different (*p* < 0.05), whereas means sharing the same superscript letter are not significantly different.

**Table 3:** Effects of nicotinamide riboside (NR) and the Nrf2 inhibitor ML385 on serum lipid profile in cisplatin-treated rats.

Group	Total Cholesterol (mg/dL)	Triglycerides (mg/dL)	LDL-C (mg/dL)	HDL-C (mg/dL)
Control	114.16 ± 17.96 ^c^	45.36 ± 3.59 ^c^	109.06 ± 8.33 ^c^	31.16 ± 2.02 ^a^
Cisplatin	216.16 ± 13.58 ^a^	177.00 ± 11.80 ^a^	198.16 ± 10.06 ^a^	19.50 ± 2.50 ^c^
Cisplatin + NR	162.83 ± 13.72 ^b^	88.67 ± 10.94 ^b^	128.83 ± 8.12 ^b^	27.83 ± 2.46 ^b^
NR	118.33 ± 11.24 ^c^	48.67 ± 4.51 ^c^	104.50 ± 7.18 ^c^	32.83 ± 2.11 ^a^
Cisplatin + ML385	206.50 ± 14.30 ^a^	166.80 ± 12.20 ^a^	188.40 ± 9.80 ^a^	20.70 ± 2.10 ^c^
Cisplatin + NR + ML385	186.20 ± 13.60 ^b^	132.40 ± 11.50 ^b^	160.50 ± 9.40 ^b^	24.60 ± 2.30 ^b^

**Table 4:** Effects of nicotinamide riboside (NR) and the Nrf2 inhibitor ML385 on hepatic oxidative stress biomarkers in cisplatin-treated rats.

Group	GSH (ng/mL)	SOD (ng/mL)	MDA (µM/L)	NO (µM/L)
Control	8.8 ± 1.1 ^a^	40.4 ± 8.1 ^a^	83.4 ± 12.2 ^c^	172.2 ± 27.2 ^c^
Cisplatin	5.3 ± 1.1 ^c^	22.8 ± 4.5 ^c^	119.6 ± 18.1 ^a^	197.3 ± 11.9 ^a^
Cisplatin + NR	7.8 ± 0.2 ^b^	34.7 ± 3.7 ^b^	99.2 ± 22.1 ^b^	182.4 ± 13.1 ^b^
NR	8.4 ± 0.9 ^a^	39.2 ± 4.3 ^a^	84.9 ± 10.8 ^c^	174.5 ± 12.6 ^c^
**Cisplatin + ML385**	**5.8 ± 0.9 ^c^**	**24.5 ± 3.8 ^c^**	**116.8 ± 16.5 ^a^**	**194.0 ± 12.3 ^a^**
**Cisplatin + NR + ML385**	**6.6 ± 0.8 ^b^^c^**	**29.2 ± 3.6 ^b^^c^**	**108.5 ± 15.0 ^a^^b^**	**188.3 ± 11.6 ^a^^b^**

**Table 5:** Effect of nicotinamide riboside (NR) on hepatic NAD^+^, NADH, and NAD^+^/NADH ratio in cisplatin-induced hepatorenal toxicity.

Group	NAD^+^ (nmol/mg Protein)	NADH (nmol/mg Protein)	NAD^+^/NADH Ratio
Control	46.3 ± 3.2 ^a^	9.2 ± 0.8 ^c^	5.03 ± 0.41 ^a^
Cisplatin	20.1 ± 2.4 ^c^	12.4 ± 1.1 ^a^	1.62 ± 0.21 ^c^
Cisplatin + NR	23.2 ± 2.3 ^c^	11.6 ± 1.2 ^a^	2.52 ± 0.25 ^c^
NR	39.7 ± 2.9 ^b^	10.3 ± 0.9 ^b^	3.86 ± 0.37 ^b^

Note: Values are expressed as mean ± SD (*n* = 6 per group). Different superscript letters (a–c) within the same column indicate statistically significant differences between groups (*p* < 0.05), as determined by one-way ANOVA. NAD^+^, nicotinamide adenine dinucleotide (oxidized form); NADH, nicotinamide adenine dinucleotide (reduced form).

**Table 6:** Effect of nicotinamide riboside (NR) and Nrf2 inhibition by ML385 on hepatic NAD^+^, NADH, and NAD^+^/NADH ratio in cisplatin-treated rats.

Group	NAD^+^ (nmol/mg Protein)	NADH (nmol/mg Protein)	NAD^+^/NADH Ratio
Control	46.3 ± 3.2 ^a^	9.2 ± 0.8 ^c^	5.03 ± 0.41 ^a^
Cisplatin	20.1 ± 2.4 ^c^	12.4 ± 1.1 ^a^	1.62 ± 0.21 ^c^
Cisplatin + NR	23.2 ± 2.3 ^b^	11.6 ± 1.2 ^b^	2.52 ± 0.25 ^b^
NR	39.7 ± 2.9 ^a^	10.3 ± 0.9 ^c^	3.86 ± 0.37 ^a^
Cisplatin + ML385	18.8 ± 2.1 ^c^	13.2 ± 1.2 ^a^	1.42 ± 0.18 ^c^
Cisplatin + NR + ML385	19.5 ± 2.0 ^c^	12.9 ± 1.1 ^a^	1.51 ± 0.20 ^c^

## Data Availability

The original contributions presented in this study are included in the article. Further inquiries can be directed to the corresponding author.

## Abbreviations

The following abbreviations are used in this manuscript:

AST	Aspartate Aminotransferase
cDNA	Complementary DNA
ELISA	Enzyme-Linked Immunosorbent Assay
GAPDH	Glyceraldehyde-3-Phosphate Dehydrogenase
GSH	Reduced Glutathione
H&E	Hematoxylin and Eosin
HPRT1	Hypoxanthine Phosphoribosyltransferase 1
KIM-1	Kidney Injury Molecule-1
MDA	Malondialdehyde
NAD+	Nicotinamide Adenine Dinucleotide
Nfe2l2 (Nrf2)	Nuclear Factor Erythroid 2-Related Factor 2
NO	Nitric Oxide
NQO1	NAD(P)H Quinone Oxidoreductase 1
NR	Nicotinamide Riboside
qRT-PCR	Quantitative Real-Time Polymerase Chain Reaction
ROS	Reactive Oxygen Species
SEM	Standard Error of the Mean
SOD2	Superoxide Dismutase 2
RT-qPCR	Reverse Transcription Quantitative Polymerase Chain Reaction
